# Current knowledge on the sensitivity of the ^68^Ga-somatostatin receptor positron emission tomography and the SUV_max_ reference range for management of pancreatic neuroendocrine tumours

**DOI:** 10.1007/s00259-016-3395-4

**Published:** 2016-05-13

**Authors:** Irene Virgolini, Michael Gabriel, Alexander Kroiss, Elisabeth von Guggenberg, Rupert Prommegger, Boris Warwitz, Bernhard Nilica, llanos Geraldo Roig, Margarida Rodrigues, Christian Uprimny

**Affiliations:** Department of Nuclear Medicine, Medical University of Innsbruck, Anichstrasse 35, 6020 Innsbruck, Austria

**Keywords:** ^68^Ga-somatostatin analogue, Neuroendocrine tumour, Pancreatic imaging, Uncinate process, Sensitivity, Specificity, SUV_max_-calculation

## Abstract

Physiologically increased pancreatic uptake at the head/uncinate process is observed in more than one-third of patients after injection of one of the three ^68^Ga-labelled octreotide-based peptides used for somatostatin (sst) receptor (r) imaging. There are minor differences between these ^68^Ga-sstr-binding peptides in the imaging setting. On ^68^Ga-sstr-imaging the physiological uptake can be diffuse or focal and usually remains stable over time. Differences in the maximal standardised uptake values (SUV_max_) reported for the normal pancreas as well as for pancreatic neuroendocrine tumour (PNET) lesions may be related to several factors, including (a) differences in the peptide binding affinities as well as differences in sstr subtype expression of pancreatic α- and β-cells, and heterogeneity / density of tumour cells, (b) differences in scanner resolution, image reconstruction techniques and acquisition protocols, (c) mostly retrospective study designs, (d) mixed patient populations, or (e) interference with medications such as treatment with long-acting sst analogues. The major limitation in most of the studies lies in the lack of histopathological confirmation of abnormal findings. There is a significant overlap between the calculated SUV_max_-values for physiological pancreas and PNET-lesions of the head/uncinate process that do not favour the use of quantitative parameters in the clinical setting. Anecdotal long-term follow-up studies have even indicated that increased uptake in the head/uncinate process still can turn out to be malignant over years of follow up. SUV_max_-data for the pancreatic body and tail are limited. Therefore, any visible focal tracer uptake in the pancreas must be considered as suspicious for malignancy irrespective of quantitative parameters. In general, sstr-PET/CT has significant implications for the management of NET patients leading to a change in treatment decision in about one-third of patients. Therefore, follow-up with ^68^Ga-sstr-PET/CT is mandatory in the clinical setting if uptake in the head/uncinate process is observed.

## Pancreas anatomy (Fig. 1) and surgery options (Fig. 2)

**Fig. 1:**
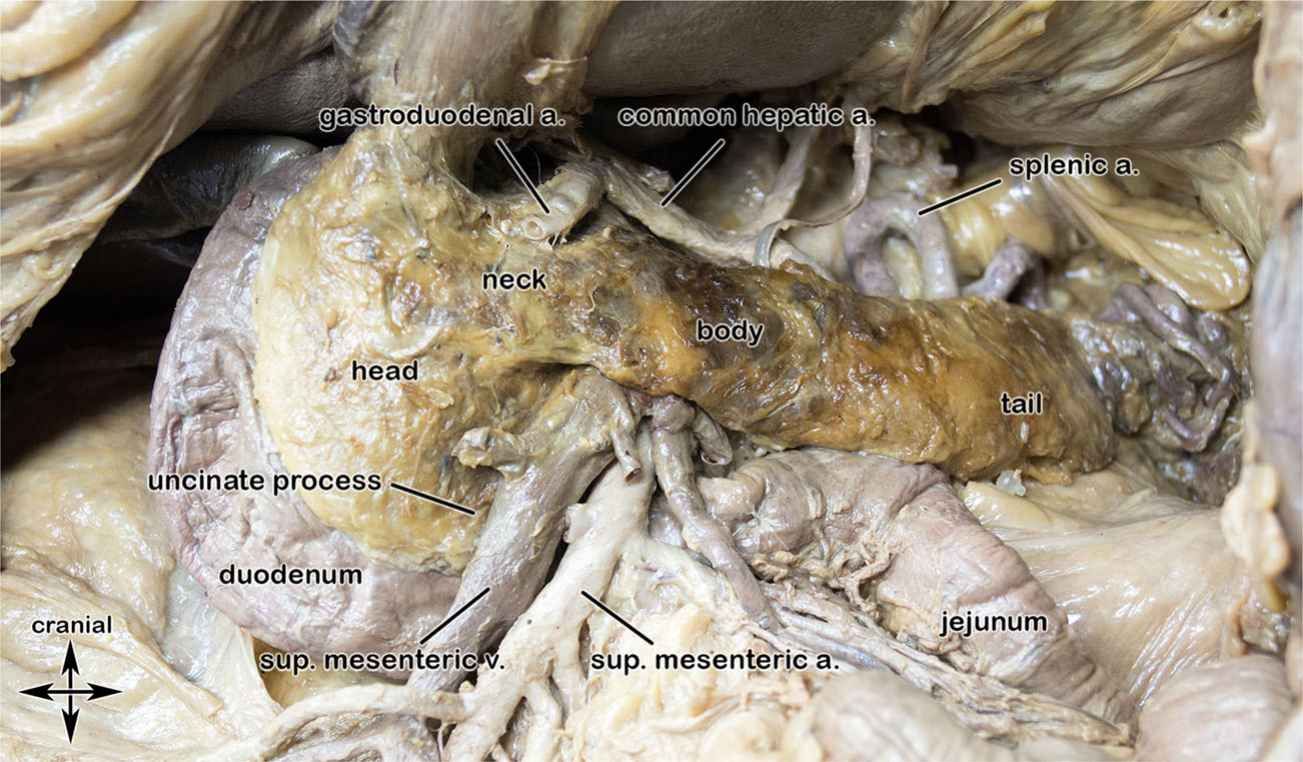
Normal pancreatic anatomy. Image obtained from a cadaveric pancreatic specimen. Courtesy of Marko Konschake, M.D., Anatomical Institute, Medical University of Innsbruck, Austria

**Fig. 2:**
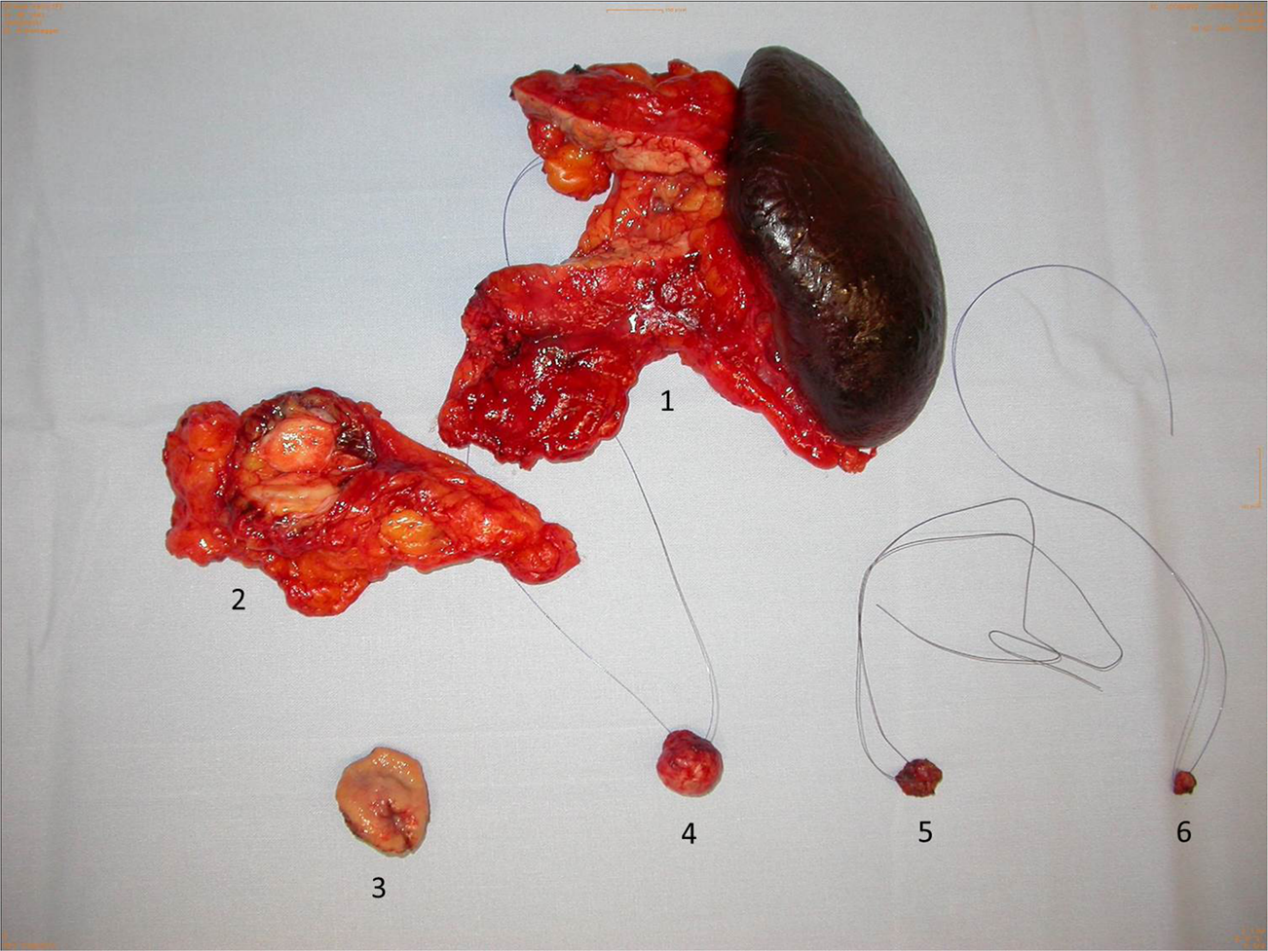
Thompson procedure. Operative specimens of multiple endocrine neoplasia (MEN) I-associated Zollinger–Ellison syndrome with hypergastrinemic-induced Type II NET of the stomach (Thompson procedure). (1) Specimen of left pancreatectomy with multiple small PNETs. (2) Metastasis of omentum majus not detected preoperatively. (3) Locally excised Type II NET of stomach induced by duodenal gastrinomas (5,6). (4) Enucleated non functioning PNET of pancreatic head. (5,6) Submucosal gastrinomas of duodenum excised by duodenotomy after transillumination

Removal of the primary tumour significantly prolongs survival of pancreatic neuroendocrine tumour (PNET) patients for both those with distant metastases and those without metastases. Therefore, early diagnosis/detection of PNETs is crucial for patient management. In general, surgery for PNETs is faced by two characteristic facts. On the one hand, preoperative diagnosis/localisation is challenging, and on the other hand, surgery of the pancreas can result in life-threatening complications. Once the diagnosis is established, choice for the optimal surgical procedure has to be made [1]. The criteria that influence this decision are the localisation of the PNET within the pancreas (caput, corpus, tail), type of PNET (i.e., insulinoma, gastrinoma, non-functioning tumours, multiple PNETs in multiple endocrine neoplasia (MEN) I syndrome), and localisation of the PNET in correlation to the pancreatic duct. Preservation of endocrine function, if at all possible, should be one consideration in the choice of operation. A radical resection should always be considered, at least for tumours of >2 cm even in cases of known distant metastases with impact on further prognosis [2]. A total of more than 60 % 5-year survival rates can be achieved after R 0-resection, independently of the extent of surgery with surgical mortality below 5 % and acceptable morbidity between 20 and 30 % in specialised centres [3]. For preoperative localisation of small PNET/duodenal wall ^68^Ga-somatostatin(sst)-receptor(r)-PET/CT showed high sensitivity for delineation of malignant lesions [4].

## Pancreas imaging modalities

There are different metabolic imaging methods, various tracers, and several anatomic modalities to stage PNETs. In principle, morphologic techniques bear the risk of underestimation. ^68^Ga-sstr-PET/CT in combination with diffusion-weighted magnetic resonance imaging (DW-MRI) is currently the most promising technique for investigating NETs. The calculated sensitivity from pooled data of 52 studies was highest for sstr-PET compared to endoscopic or intraoperative ultrasound, sstr-scintigraphy, dual-phase (DP)-CT, DW-MRI, and ^18^F-FDG-PET, according to a recent extensive review by Bodei et al [5]. In a small number of patients, other molecular PET tracers such as ^18^F-DOPA, ^11^C-5-HTP [6] or the ^68^Ga-exendin-4 targeting glucagon-like peptide-1 (GLP-1) [7] receptor tracer may locate a lesion when sstr-PET/CT is negative.

## Basis of pancreatic imaging with sstr-ligands

### Expression of sstr subtypes on normal pancreatic and on pancreatic neuroendocrine tumour cells

For PNETs peptide receptor imaging has been a challenge over the last 20 years, and still is. In principal, NET cells may express much higher amounts of sstr than normal tissue or blood cells providing the basis for imaging and treatment of sstr-positive tumour lesions. However, different primary tumours and metastases may express different amounts as well as different types of sstr-subtypes, and peptide ligands may bind differently to the five sstr-subtypes known [8, 9].

In fact, not too much is known about the normal in-vivo expression of sstr—and there may be large inter-individual variation as well. Physiologically, several normal organs show an increased sstr-expression. Therefore, different organs including the spleen, liver, pituitary, thyroid, kidneys, adrenal glands, salivary glands, and “the pancreas” may be visualised by ^68^Ga-sstr-PET-imaging.

Usually this physiologic pancreatic uptake appears as a “faint” uptake along the head, body, and tail of the pancreas that is believed to be a result of binding with high affinity to pancreatic islet cells that express various sstr subtypes. Pancreatic polypeptide (PP) cells are not distributed equally in the gland; they are the most abundant cell type in the posterior part of the pancreatic head whereas they are scarce or absent in the remainder of the gland. This lobe probably originates from the ventral pancreatic bud during embryogenesis [10]. Variability in the normal expression of sstr1-5 subtypes among the different human endocrine pancreatic cells was shown by Portela-Gomes et al [11]. Sst generally exerts inhibitory effects on hormone release produced in the pancreatic islets [12] mediated through the five known G protein-coupled sstr subtypes [13].

### Sstr binding affinity of ^68^Ga-sstr-ligands

Different sst-analogues have been introduced for ^68^Ga-sstr-PET-imaging over the past 10 years, with the first PET-imaging studies in humans performed with ^68^Ga- 1,4,7,10-tetraazacyclododecane-1,4,7,10-tetraacetic acid (DOTA)- Tyr3-octreotide (TOC) [14]. The small structural change achieved by introducing the metal gallium instead of other trivalent metals (indium, yttrium, lutetium) appeared to impact the affinity profile for the different sstr-subtypes (Table 1, [15–18]). Furthermore, the pharmacokinetic properties also showed improvement [15]. The Ga-complexes of sst-analogues commonly show a higher binding affinity for sstr2 when compared with the corresponding complexes with indium, yttrium, or lutetium. The increased hydrophilicity of the Ga-complex furthermore results in an increased renal elimination. Together with an improved accumulation of ^68^Ga-DOTA-TOC in the tumour lesions these pharmacokinetic properties lead to a high lesion contrast within a short time interval post injection, which is of particular importance considering the short half-life of ^68^Ga (68 min). Ga-DOTA- Tyr3-octreotate (TATE) with a C-terminal threonine instead of the corresponding amino alcohol in DOTA-TOC shows a 10-fold higher affinity towards sstr2, whereas the affinity to sstr3 and sstr5 is reduced and the affinity to sstr4 is increased when compared with Ga-DOTA-TOC. Ga-DOTA- 1-Nal3-octreotide (NOC) with tyrosine in position 3 replaced for 1-naphtyl-alanine shows a similar sstr2-binding affinity together with a more than 10-fold increased affinity for sstr3 and sstr5 in comparison to Ga-DOTA-TOC and a similar affinity to sstr4 when compared to Ga-DOTA-TATE [16].

**Table 1:** Affinity profiles of sst analogues

Peptide	sstr1	sstr2	sstr3	sstr4	sstr5
DOTATOC	>10,000	14 ± 2.6	880 ± 324	>1,000	393 ± 84
DOTATATE	>10,000	1.5 ± 0.4	>1,000	453 ± 176	547 ± 160
DOTALAN	>10,000	26 ± 3.4	771 ± 229	>10,000	73 ± 12
Ga-DOTATOC	>10,000	2.5 ± 0.5	613 ± 140	>1,000	73 ± 21
Ga-DOTATATE	>10,000	0.2 ± 0.04	>1,000	300 ± 140	377 ± 18
Ga-DOTANOC	>10,000	1.9 ± 0.4	40.0 ± 5.8	260 ± 74	7.2 ± 1.6
Y-DOTATOC	>10,000	11 ± 1.7	389 ± 135	>10,000	114 ± 29
Y-DOTATATE	>10,000	1.6 ± 0.4	>1,000	523 ± 239	187 ± 50
Y-DOTANOC	>1,000	3.3 ± 0.2	26 ± 1.9	>1,000	10.4 ± 1.6
Y-DOTALAN	>10,000	22.8 ± 4.9	290 ± 105	>10,000	16.3 ± 3.4

The table lists the values for the inhibitory constant (nmol/L) for sstr-binding peptides and their gallium and yttrium complexes. The IC_50_-value indicates the concentration when 50 % of binding is inhibited

## Pancreatic sstr-imaging—sensitivity, specificity, and SUV_max_ (Table 2)

**Table 2:** Overview of clinical imaging studies with ^68^Ga-Sstr-analogues in PNETs

Radiopharmaceutical	Authors (Ref.)	Design	No of pts.	Quantitative parameters (SUV_max_)	Diagnostic performance (Sens./Specif.)	“Gold”/Reference standard
^68^Ga-DOTA-TOC	Gabriel et al. (20)	Prospective	84	Not done	Sensitivity 100 %, Specificity 88.8 %*	Follow-up/Histopathology
	Gabriel et al. (21)	Retrospective	46	Mean 43.6 (range 20.5–94)	N ot done	Follow-up
	Kroiss et al. (22)	Retrospective	249	Cut-off 17.1 (malignant/non-malignant)	Sensitivity 90 %, Specificity 93.6 %	Follow-up
			311 studies	6.5 ± 2.5 (range 1.6–20.7) pancreatic body		
			311 studies	10.5 ± 4.1 (range 2.9–28.7) uncinate process		
			30	Mean 33.6 ± 14.3 PNET uncinate process		
			13	Mean 36.3 ± 21.5 PNET pancreatic tail		
	Al-Ibraheem et al. (23)	Retrospective	43	Pancreatic to liver ratio range 3.24–9.1	Not done	Follow-up
			23	4.0 ± 0.8 (range 2.4–5.3) no visible uptake		
			20	9.3 ± 3.1 (range 5.1–15.5) no pathology		
			3/20	51.6 ± 15.7 (range 34.1–64.2) PNET		Histopathology
	Kumar et al. (24)	Retrospective	20	Median 12.6 (range 8.8–27.6)	Sensitivity 100 %	Follow-up/Histopathology
	Jacobsson et al. (26)	Retrospective	50	Mean 9.2 ± 2.9 (without pathology)	Not done	Concomitant CT
^68^Ga-DOTA-NOC	Prasad V, Baum RP (25)	Retrospective	50	Mean 5.8 ± 2.0 (range 4–9.7) non-malignant	Not done	Follow-up
			26	Mean 20.8 ± 10.8 PNET		
				Cut off 8.6 malignant/non-malignant		
	Castellucci et al. (27)	Retrospective	100	Mean 12.6 ± 2.2 non-malignant	Not done	Follow-up
	Prasad et al. (28)	Retrospective	59	Mean 18.6 ± 9.8 (range 7.8–34.8) previously unknown PNET	Not done	Follow-up
				Mean 26.1 ± 14.5 (range 8.7–42.4) previously known PNET		
	Krausz et al. (29)	Retrospective	96	(Range 4.7–165) suspected PNET	Not done	Follow-up
			35	Mean 25.7 ± 28.8 (range 5.5–165) PNET		
	Sharma et al. (30)	Retrospective	141	Mean 14.7 ± 6 (range 5–32.5)	Sensitivity 85.7 %, Specificity 79.1 %	Follow-up/Histopathology
^68^Ga-DOTA-TATE	Wild et al. (33)	Retrospective	8	Median 3.5 (range 3.0–4.3)	Not done	Follow-up/Histopathology
	Skoura et al. (34)	Retrospective	728	Not done	Sensitivity 97 %, Specificity 95.1 %^§^	Follow-up
	Kunikowska et al. (36)	Retrospective	250	Mean 9.2 ± 3.3 without pathology in 41/250 pts (16.4 %)	Not done	Follow-up
	Haug et al. (37)	Retrospective	104	Not done	Sensitivity 81 % %, Specificity 90 %&	8 of 36 histologically verified tumours were in the pancreas
	Schmid-Tannwald et al. (39)	Retrospective	18	Mean 36.46 ± 27.38 (range 6.2 to 120)	Not done	Histopathology
	Etchebehere et al. (40)	Prospective	19	Not done	Sensitivity 100 %, Specificity 80 %	Follow-up/Histopathology
					*with regard to patients with suspected NETs	
					^§^14 FP in 1258 scans, 4 of them for the pancreas	
					&7 FP and 7 FN, sites of findings not indicated	

Sstr-PET is nowadays the established standard method for molecular imaging of NET. A detailed procedure guideline for PET/CT with ^68^Ga-sst-analogue imaging was summarised by the Oncology Committee of the EANM [19] This (early) guideline already mentions that physiological focal tracer uptake may mimic tumour disease in the pancreas, most frequently in the head/uncinate process, and also strongly recommends anatomical localisation with other imaging modalities.

Here, we overview the currently available data on the diagnostic performance of ^68^Ga-sstr-PET in the detection of PNETs. We understand that patients at various centres occasionally have undergone surgery for suspected PNETs visualised by sst analogs in which no tumours were ultimately identified by pathohistology.

### Data for ^68^Ga-DOTA-TOC-PET

**Fig. 3:**
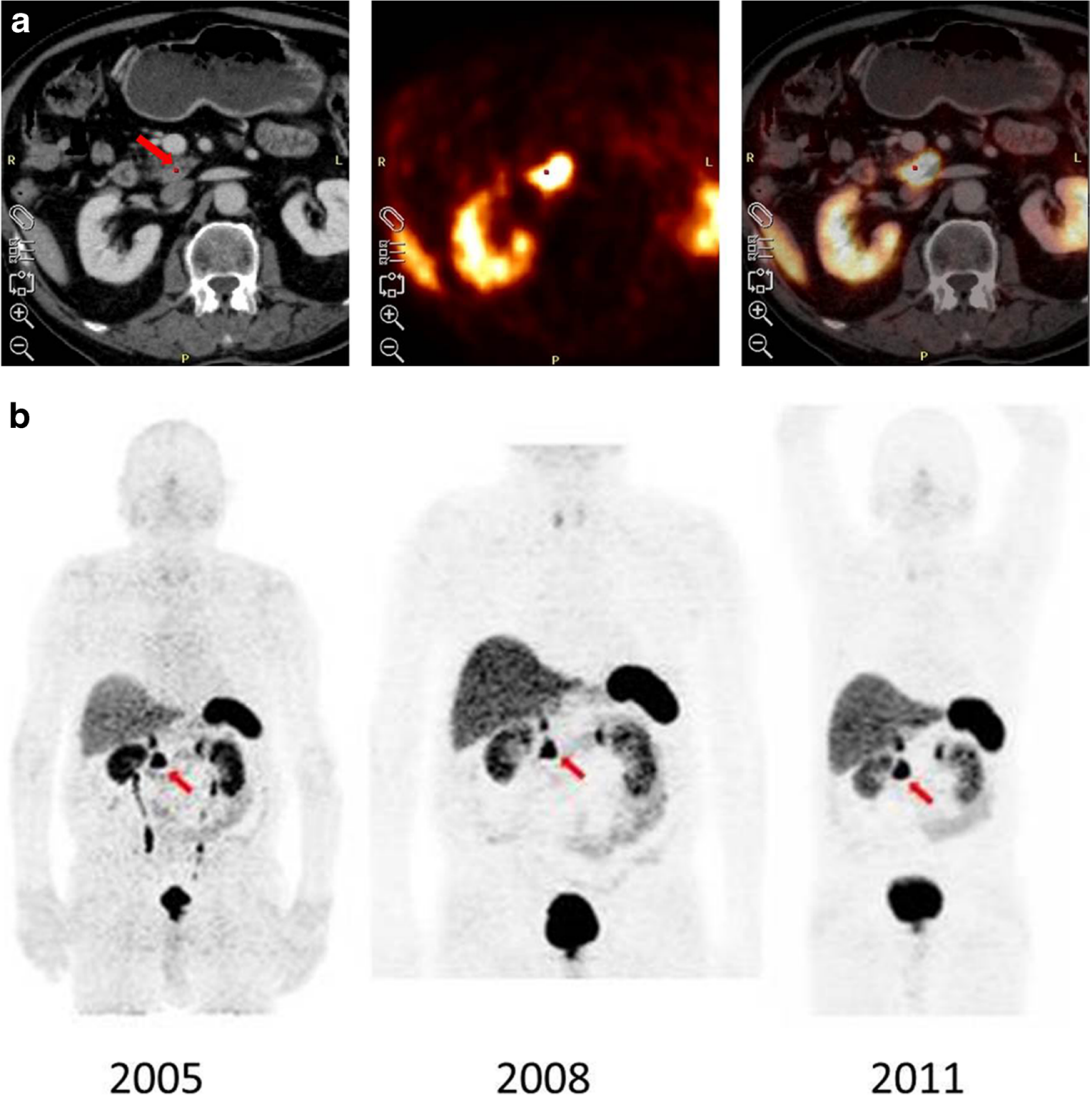
Patient with “false positive” ^68^Ga-DOTA-TOC uptake. **a** ^68^Ga-DOTA-TOC PET of patient S.J. before surgery in 2004. The images clearly indicate significant accumulation in the pancreatic head/uncinate. **b** ^68^Ga-DOTA-TOC PET (MIP) of patient S.J. after surgery in 2005, 2008, 2011. The images indicate stable accumulation in the follow-up period. Despite the significant accumulation in the pancreatic head/uncinate process, surgical exploration gave a benign histology and further follow-up studies with either PET or CT did not confirm a malignant finding. Therefore, this finding was considered a “FP” PET-result in the follow-up for the publication in 2007 [20]. The patient had further follow-up PET/CT scans performed in 2008 and 2011 that were both positive again for the same location. The SUV_max_ calculated for the uncinate process was 26.9 in 2008 and 26.5 in 2011, showing that the SUV_max_ remained stable over time, though the tumour markers chromogranin A (CGA) and neuron specific enolase (NSE) remained elevated in 2011. This patient is still alive in 12/2015 and in good clinical condition

In the first prospective publication [20] in 84 patients with known or suspected NET we described an increased uptake in the pancreatic head in 57 (67.8 %)/84 patients, but we did not quantify the uptake in terms of SUV_max._. The criteria for visual study interpretation involved a malignant pancreatic head: an irregular or protrusive shape of finding and clear delineation from adjacent tissue with higher uptake than the liver. By these criteria, PET identified 23 findings in the pancreas while there were only 21 identified with SPECT and 19 with CT. Only one false positive (FP) finding with an enhanced tracer uptake in the pancreatic head was found in a patient who had clinical symptoms in terms of persistent diarrhoea suggestive for a PNET (Fig. 3).

One of the first papers on SUV_max_-calculation [21] indicated that individual sstr-mediated tumour uptake shows a large range of variation. In this retrospective study the SUV_max_-values for tumours were ranging between 6.4 and 267, but not all tumours of this study were NETs. The study was aimed at evaluating if calculation would predict a response to peptide receptor radionuclide therapy (PRRT). In seven patients, malignant lesions of the pancreas were used for assessment of treatment response. These seven patients showed high SUV_max_-values upon the initial visit (mean 43.6; range 20.5–94). Although the reference standard (clinical follow-up by CT or MRI) indicated progressive disease (PD) in four patients and stable disease (SD) in three patients, a significant decline of overall SUV_max_-values was observed with regard to the pancreatic lesion (mean 28.9; range 18–28.9) after finalisation of PRRT. It is open for discussion as to whether the decline of tracer uptake despite PD presents a partial therapy success in terms of mixed response or whether it indicates a further step towards dedifferentiation at a cellular level.

In order to evaluate and establish the ^68^Ga-DOTA-TOC uptake for PNET lesions and differentiate the physiologic pancreatic uptake, our group calculated the SUV_max_ [22]. The SUV_max_ amounted to 34.6 ± 17.1 for PNET lesions (*n* = 43). We differentiated these lesions and calculated 33.6 ± 14.3 for tumours of the uncinate process (*n* = 30) and 36.3 ± 21.5 for the pancreatic tail (*n* = 13). The pancreatic body served as “normal pancreas” for calculating the ratios. “Normal” pancreas was defined as faint/no uptake. Finally, a mean cut-off SUV_max_-value of 17.1 was assessed for differentiating tumours in the uncinate process with a specificity of 93.6 % and a sensitivity of 90 %. At this cut-off value we found true positive (TP) = 27, FP = 20, false negative (FN) = 3, true negative (TN) = 290, and the three cases of FN (i.e., CT-positive) with SUV_max_-values of 14.7, 12.8, and 11.7. We concluded that physiological uptake can pose difficulties in correctly analysing this region. In our cohort of >1500 ^68^Ga-DOTA-TOC-PET/CT scans (years 2010–2014), 115 studies were performed to detect or exclude pancreatic malignancy (unpublished data): for 14 patients who underwent subsequent surgery, the SUV_max_-data of histopathologically verified PNETs indicated large variation, for the uncinate process 33.2 ± 15.1 (*n* = 3), pancreatic body 57.9 ± 67.6 (*n* = 3), and the pancreatic tail 47.2 ± 39.7 (*n* = 9). Cut-offs obtained by ROC (Receiver Operating Characteristic) analyses were 17.1 for the uncinate process (specificity 92 %; sensitivity 100 %), for the pancreatic body 14.4 (specificity 100 %; sensitivity 100 %) and for the pancreatic tail 12.0 (specificity 92.5; sensitivity 88.9 %).

Al-Ibraheem et al. [23]. reported no visual uptake in the pancreatic head in 20 of 43 (46.6 %) consecutive patients . In the other 23 patients (53.4 %) either focal uptake (*n* = 10) or an irregular uptake pattern (*n* = 13) were observed. The SUV_max_ for the 20 patients without visual uptake amounted to 4.0 ± 0.8 (range 2.4 to 5.3). For another 20 patients with noticeable uptake without malignancy (eight patients with focal uptake, 12 patients with irregular uptake) the SUV_max_ amounted to 9.3 ± 3.1 (range 5.1 to 15.5). In subsequent studies, malignancy could only be histologically proven in three patients (two with focal and one with irregular uptake) and the SUV_max_ in these patients amounted to 51.6 ± 15.7 (range 34.1 to 64.2). The authors concluded that if the uptake in the pancreatic head is similar to the uptake in the liver, then a physiological condition is most likely, but also noted that misalignment due to respiratory motion must always be taken into account.

Kumar et al. [24] found 100 % sensitivity for detecting primary pancreatic tumours as well as metastatic disease and reported that the detection rate of PET/CT was higher compared to CT alone, both for primary tumours (20 versus 15) as well as metastases (13 versus seven). They reported no significant correlation of SUV_max_-values with tumour size. Primary tumours were localised in the pancreatic head in seven, in the body in nine and in the tail in three patients. Median SUV_max_ of primary tumours amounted to 12.6 (range 8.8 to 27.6), similar to the values reported by Prasad and Baum [25] for ^68^Ga-DOTA-NOC. In Fig. 2 they show a case of significant uptake in the pancreatic head on PET/CT, which was later verified by pathohistology to be a well-differentiated NET lesion (SUV_max_ 12.6; personal communication). The authors state that there was no FP for primary PNETs and that all tumours were seen on ^68^Ga-DOTA-TOC-PET.

Jacobsson et al. [26] retrospectively analysed their cohort and reported physiological ^68^Ga-DOTA-TOC-uptake in the uncinate process in 35/50 (70 %) patients. These authors calculated a mean SUV_max_ of 9.2 ± 2.9 for the uncinate process using an isoactivity cut-off of >75 % and >50 % that equated to 7.8 and 6.0, respectively. The uncinate process activity versus pancreas body and tail varied between 3.0 and 4.3.

### Data for ^68^Ga-DOTA-NOC-PET

Prasad and Baum [25] reported an SUV_max_-value of 5.8 ± 2.0 (range 4–9.7) for the physiological uptake of the uncinate process (*n* = 50) and significantly higher SUV_max_-values for PNETs (20.8 ± 10.8; *n* = 26), providing high target to non-target ratios. The authors suggested that the uncinate process can be differentiated from pancreatic tumours with high diagnostic accuracy at an SUV_max_-cut-off of 8.6 (sensitivity 92 %, specificity 94 %).

Castellucci et al. [27] retrospectively assessed 100 patients and identified 23 % diffuse and 8 % focally increased pancreatic tracer uptake in patients with extra-pancreatic NETs in whom follow-up PET indicated no change of SUV_max_-values over time, thus suggesting no disease. The SUV_max_ was 12.6 ± 2.2 for those with focal uptake and 5.0 ± 1.6 for diffuse uptake. The values refer to uptake in the head/uncinate process without known pathology albeit no histology was obtained. The authors state that the pancreatic pattern of uptake remained unchanged, regardless of whether it was focal, diffuse, or absent. They could not confirm the low cut-off value suggested by the Bad Berka group [20] or come close to our Innsbruck suggestion that the mean SUV_max_-cut-off between benign and malignant pancreatic tissues should lie somewhat “higher”.

However, in a bi-centric study [28] in 59 NET-patients with unknown primary PNETs, PET/CT were able to localise the tumours in 35/59 (59 %) patients, including 16 patients with PNETs (five head, four body, three tail, three multifocal, and one uncinate process). Notably, primary PNETs found on CT after retrospective analyses were reported in 8/16 patients (50 %)! The mean SUV_max_ of previously identified unknown primary PNETs was 18.6 ± 9.8 (range 7.8–34.8) and the mean SUV_max_ in patients with a previously known PNET was 26.1 ± 14.5 (range 8.7–42.4), suggesting that patients with known primary PNETs have higher sstr expression. Notably, the authors present a case of multifocal PNET in Fig. 2a of their study that was only detected by PET/CT at the pancreatic tail but was missed by endoscopic ultrasound (EUS). Furthermore, the average time interval between the first biopsy-proven diagnoses of NET and the first evidence of a primary tumour on PET/CT was found to be approximately 29 months!

Krausz et al. [29] reported for 35 PNETs an SUV_max_ ranging between 5.5 and 165 (mean 25.7 ± 28.8). Among 63 cases without previously known pathology, uptake was suspicious for tumour in 24 cases with an SUV_max_ ranging between 4.7 and 35 (mean 16.3 ± 8.0). In 38 sites they judged this uptake as physiological, generally lower relative to adjacent structures (SUV_max_ 2.2–12.6; mean: 6.6 ± 2.2). In 24 scans with suspected tumour and in 37/38 scans with physiological uptake, diagnostic CT/MRI/EUS failed to detect tumour, suggesting that these investigations do not significantly add to a diagnosis if uptake is observed in the pancreatic uncinate. No ROC analysis is available because of low specificity and sensitivity rates.

Sharma et al. [30] reported a mean overall SUV_max_ for primary PNETs of 14.7 ± 6 (range 5–32.5), which was significantly higher than that of metastases overall (*P* = 0.001), as well as in the diagnosis/staging (*P* = 0.041) and restaging (*P* = 0.0003) subgroups. For the staging/diagnosis group (*n* = 88), the overall sensitivity was 73 %, specificity was 50 %, and accuracy was 70.4 %, and these values were significantly lower (*p* < 0.0001) when compared to the restaging group (98.6, 100, 98.8 %, respectively). Of the 88 studies, 57 were TP, five were TN, five were FP and 21 were FN, and of the latter ones most of these were known or suspected insulinomas. In the non-insulinoma group the sensitivity was 100 %, specificity was 57.1 %, and accuracy was 94.8 %.

### Data for ^68^Ga-DOTA-TATE-PET

Poeppel et al. [31] reported in 40 patients (head-to-head comparison) with metastasised NETs that significantly fewer lesions were detected by ^68^Ga-DOTA-TATE as compared to ^68^Ga-DOTA-TOC (*p* < 0.05, 254 vs. 262 lesions), but no overall difference was found for detection of regions (78 vs. 79 regions were positive). However, the SUV_max_-values were significantly lower for ^68^Ga-DOTA-TATE compared to ^68^Ga-DOTA-TOC (*p* < 0.01; 16.0 ± 10.08 vs. 20.4 ± 14.7) across all lesions including those of the pancreas.

In contrast, Kabasakal et al. [32] reported in a head-to-head comparative study a comparable diagnostic accuracy for both sstr peptides in 20 tumour patients on a visual scale. However, ^68^Ga-DOTA-TATE identified 14 more tumour lesions because of superior uptake compared to ^68^Ga-DOTA-NOC. In fact, the SUV_max_-values calculated for the lesions were significantly higher, with 29.9 ± 26.4 for ^68^Ga-DOTA-TATE compared to 24.5 ± 20.3 for ^68^Ga-DOTA-NOC (*p* < 0.001). Pancreatic uptake was not further classified in this paper.

A prospective study from London [33] performed in 18 patients reported a lesion-based overall sensitivity of 93.5 % for ^68^Ga-DOTA-NOC compared to 85.5 % for ^68^Ga-DOTA-TATE, which was due to a significantly (*p* < 0.001) higher detection of liver metastases. As for PNETs, ^68^Ga-DOTA-NOC detected 7/8 whereas ^68^Ga-DOTA-TATE detected only 3/8 lesions. In two patients with MEN 1 ^68^Ga-DOTA-TATE detected only one PNET whereas ^68^Ga-DOTA-NOC detected multiple PNETs. Both tracers detected an uncinate process lesion in two patients that were not confirmed by cross-sectional and follow-up imaging and were therefore regarded as FP. The SUV_max_-values for the physiological pancreas were significantly (*p* < 0.001) higher for ^68^Ga-DOTA-TATE as compared to ^68^Ga-DOTA-NOC (3.5, 3.0–4.3 vs 2.5, 2.0–3.4). The same group also reviewed their cohort of 728 patients and found 14 FP scans out of 1258, four of them for the pancreas, with an overall sensitivity of 97 % and specificity of 95.1 %, and change in treatment management in 41 % of patients owing mainly to new unexpected findings [34].

**Fig. 4:**
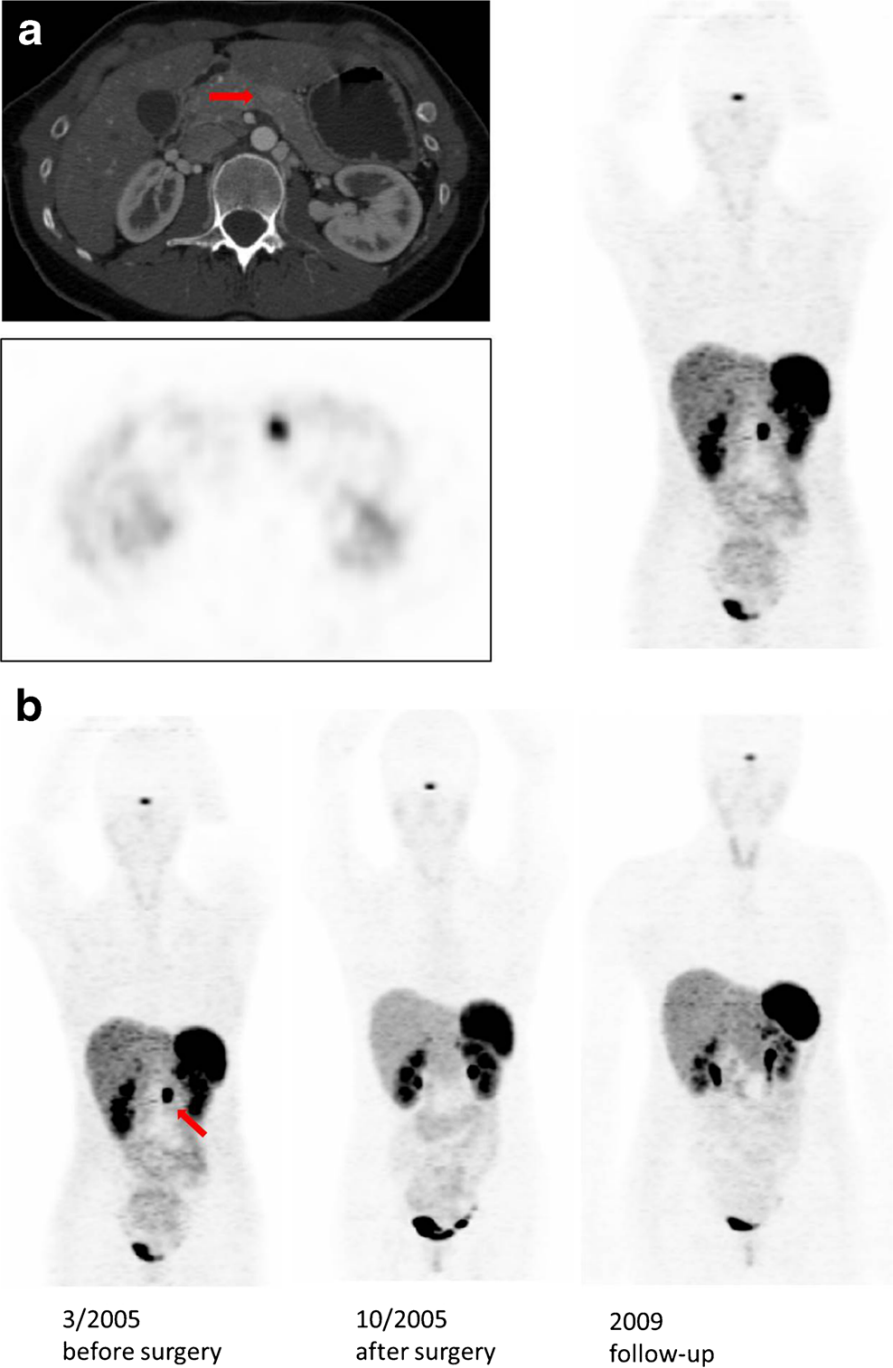
Patient with “true positive” ^68^Ga-DOTA-TOC uptake. **a** ^68^Ga-DOTA-TOC PET of patient R.R. before surgery in 2005. The images clearly indicate significant accumulation in the pancreatic body. **b** ^68^Ga-DOTA-TOC PET (MIP) of patient R.R. before and after surgery in 2005 and 2009. The images indicate no tumour recurrence. This female patient [20] aged 28 years had a 1-cm hypodense lesion on CT as well as an elevated uron specific enolase (NSE) and chromogranin A (CGA), and underwent surgery. Histology was a well-differentiated PNET and no metastases were found in the follow-up PET/CT scans in 2005 and 2009. The SUV_max_ of the lesion was 15

In a retrospective evaluation Hofman et al. [35] communicated a high impact on patient management, including curative surgery, by identifying a primary site and directing patients with multiple metastases to systemic therapy. Uncinate process uptake was generally not seen on ^111^In-octreotide imaging but was visualised in 32 % (*n* = 19) by PET. One FP case was due to moderately increased uptake in the uncinate process that appeared particularly prominent. This case was concordant with a previous ^111^In-octreotide scan and was performed to exclude additional disease. The patient underwent surgery and histology revealed no evidence of a NET, although the referrer marked that the patient’s symptoms and biochemistry improved. They also demonstrate the case of a previously unknown PNET in Fig. 4.

Kunikowska et al. [36] reported increased physiological uptake in the uncinate process in 41/250 patients (16.4 %) with an SUV_max_ of 9.2 ± 3.3. In this study, sites of physiological focal uptake were confirmed to be disease-free in subsequent follow-up imaging studies as well as in clinical follow-up. The authors also reported in one patient a 1-cm lesion that was only detected by DW-MRI, and subsequent surgical resection revealed an adenocarcinoma with hyperplasia of neuroendocrine cells (Fig. 8 of their paper, SUV_max_ 7.2; personal communication).

Haug et al. [37] indicated a sensitivity of 81 % and a specificity of 90 %, resulting in an accuracy of 87 % with seven FN and seven other FP cases in suspected NETs. They also reported a change in surgical management in 9/44 (20 %) of patients; among those were 6/18 (33 %) patients with PNETs [38].

Schmid-Tannwald et al. [39] showed that ^68^Ga-DOTA-TATE-PET/CT is more sensitive than DW-MRI in the detection of PNETs in 18 patients in the first direct head-to-head comparative study of patients with histologically proven well-differentiated or intermediate PNETs. The cohort comprised five lesions in the head, three in the uncinate process, six in the body and nine in the tail. In this study, the SUV_max_ for the PNETs were 36.46 ± 27.38, range 6.2–120. In Fig. 4 they show the case of a patient with PNET that could neither be visualised by DW-MRI nor CT. Only the fused PET/CT image made the lesion clearly identifiable by its increased uptake of ^68^Ga-DOTA-TATE.

Similarly, Etchebehere et al. [40] reported for 19 patients a higher sensitivity of PET/CT over DW-MRI (1.5 T) and SPECT/CT (^99m^Tc- 6-hydrazinonicotinic acid (HYNIC)-octreotide). This study was performed prospectively and included a biopsy of suggestive lesions. For the pancreas, PET/CT detected more lesions versus SPECT/CT (*P* = 0.0455) and WB-DWI (*p* < 0.0455) including a case of unknown primary in the pancreas uncinate (sensitivity 100 %, specificity 80 %, accuracy 84 %), but also reported on one FP-uptake in the process uncinate. Notably, in Fig. 1 the authors demonstrate the case of an unknown primary which was clearly marked only on ^68^Ga-DOTA-TATE-PET/CT while neither SPECT/CT nor MRI identified the tumour. Only retrospective analysis detected the primary (0.4 cm lesion) on dedicated CT performed 4 years previously!

### Data for other sstr-binding radiopharmaceuticals

#### ^68^Ga-DOTA- lanreotide (LAN) and ^64^Cu-DOTA-TATE-PET

The only PNET imaged by ^68^Ga-DOTA-LAN, which was negative by ^68^Ga-DOTA-TOC, exhibited an SUV_max_ of 6.5 [41]. Pfeifer et al. [42] reported a case of previously unknown PNET that was only confirmed 6 months later on CT in a patient with MEN I syndrome.

## Accuracy of SUV_max_-determination and limitation

Sstr targeting is demonstrated by visualisation of tumour lesions. This visualisation is based on an increased expression of sstr by tumour lesions [8, 9]. There is a “visual scale” introduced initially by the group of Krenning et al. [43], but several other groups have also tried to quantify the sst tracer uptake. This can be (semi) quantified by SUV_max_ or dosimetry calculation and qualifies a patient as being sstr-positive on a scan with potential for PRRT [44]. Several factors may interfere with SUV_max_-calculation.

### Sstr-binding affinity

The discussion was raised that the (slightly) different sstr2-binding affinities for ^68^Ga-DOTA-TOC, ^68^Ga-DOTA-NOC, and ^68^Ga-DOTA-TATE may result in a somewhat different SUV_max_ for tumours and probably also for the physiologic uptake. Direct comparative head-to-head PET/CT data were published only in few numbers of patients [31–33]. Due to the 10-fold higher binding affinity for the sstr2 subtype, DOTA-TATE may theoretically bear the potential for better tumour detection. Interestingly, the comparative study of ^68^Ga-DOTA-TOC and ^68^Ga-DOTA-TATE exhibited a higher tumour uptake for ^68^Ga-DOTA-TOC [31]. The higher affinity of ^68^Ga-DOTA-TATE to sstr2 could be contra-balanced by the higher affinity of ^68^Ga-DOTA-TOC to sstr5. The two radioligands showed, however, a high inter- and intra-patient variability and possessed a comparable diagnostic value for the detection of NET lesions. Potential advantages of ^68^Ga-DOTA-TOC are the superior SUV_max_ derived for tumour lesions and the higher tumour-to-kidney ratio. For ^68^Ga-DOTA-NOC a better visualisation of normal organs, especially the spleen, was reported because of the broader subtype affinity profile [45]. ^68^Ga-DOTA-NOC showed a lower normal liver and pancreas uptake with respect to ^68^Ga-DOTA-TATE, allowing for a higher detection rate of liver metastases and PNETs. On the other hand, ^68^Ga-DOTA-TATE showed a higher uptake in organs with predominant sstr2 expression and in bone metastases than ^68^Ga-DOTA-NOC. ^68^Ga-DOTA-NOC PET was shown to be superior in the detection of PNETs, however, with both tracers FP results for the pancreatic uncinate were reported [33]. Similar FP findings have been reported for ^68^Ga-DOTA-TOC and ^68^Ga-DOTA-NOC by others [22, 27].

Uptake in normal pancreas is thought to be based on the presence of sstr on the islet cells [46]. High concentration of sstr2 has been consistently found on pancreatic α- and β-cells but sstr3 and sstr5 concentration is still controversial. This normal expression may increase the risk of FP findings. However, Wittingen and Frey [47] demonstrated that the islet concentration in the tail is greater as compared with the uncinate head/body, which does not support this hypothesis. The controversial expression of sstr may be one reason for the considerable overlap of the values reported in the literature, though physiological uptake in the pancreas was generally associated with lower SUV_max_-values compared to tumour lesions. Furthermore, the calculated SUV_max_-values for PNETs may have a broad range of variation as indicated in Table 2. Whereas Miederer et al. [48] found a positive correlation of SUV_max_ with the sstr2 expression using ^68^Ga-DOTA-TOC-PET/CT, there is some evidence that at an SUV_max_ greater than 25 the calculation may not reflect solely sstr expression of tumours [49].

### Scanner resolution and image reconstruction techniques

The tomographic resolution of a PET scanner and the method of data reconstruction significantly affect SUV_max_-calculation, especially for small NET lesions with high uptake. In a comparison of standardised protocols with 23 dedicated PET scanners Geworski et al. [50] demonstrated errors of up to 10 % in SUV_max_-values. Furthermore, many centres were not able to maintain accurate SUV_max_-calibrations without additional supervision, which can be another hindrance of reasonable SUV_max_-calculation [51].

Time of flight (TOF) is a valuable method for acquiring the signal-to-noise ratio [52]. Basically, TOF generates an increase in SUV_max_. Therefore, the use of TOF has to be taken into account when comparing SUV values.

In recent years new reconstruction algorithms have continuously been developed that mainly confirm under the notation “resolution recovery”. These improving algorithms take known scanner, bed, and physical geometries into account and vary from vendor to vendor. On the one hand they may enhance the signal-to-contrast ratios, and on the other hand, they may also reduce the activity administered to the patient or reduce the time per bed position. The common hurdles to introducing these patient exposure-reducing and image-improving algorithms quickly into daily routine are the significant costs, the need for physician training and familiarity with modified images, and the strongly reduced possibility to use the different reconstructed images for follow-up comparison. Therefore, the PET scanner scene is very heterogenic in terms of technological standard (e.g., TOF or nonTOF) and the use of modern reconstruction algorithms. This makes it almost unreliable in comparison of SUV_max_-values from sites with different scanners.

The Innsbruck data with ^68^Ga-DOTA-TOC [22] were derived with the use of TOF and geometric modelling (VuePoint FX, GE®), contrary to the reconstruction methods used in the studies reported by Prasad and Baum [25] with ^68^Ga-DOTA-NOC and Poeppel et al. [31] with ^68^Ga-DOTA-TATE.

### Other factors

#### Tracer uptake time

The initial patient study [20] clearly demonstrated that serial acquisition at different time points (20, 60, and 100 min p.i.) provide different results with regard to image quality and count ratios. Based on the pharmacokinetics on one hand and on the rapid isotope decay on the other, the optimal time point for scanning should be 100 min p.i.. However, time points for scanning are still not standardized [43], but would be an important methodological requirement for comparison of semi-quantitative parameters.

#### Peptide mass

Some evidence suggests that the peptide mass may potentially influence the in-vivo binding and subsequently SUV_max_ [53], but there exist no further data on this hypothesis.

#### Motion

Difficulties in image interpretation may also stem from respiratory motion contributing to FP pancreatic uptake. Therefore, attenuation correction is essential [54].

#### Influence of PRRT and other therapies

Receptor downregulation may become a problem under different therapy modalities and may also change receptor density due to dedifferentiation in the course of disease [45].

## Impact for patient management

PNETs can present with various symptoms and clinical outcomes. Symptomatic tumours include insulinomas, gastrinomas, glucagonomas, VIPomas, and somatostatinomas, but most of the PNETs are non-functioning and present fairly late, usually with liver metastases. Thus, early and accurate localisation of primary tumours and metastases is essential for further patient management.

Hyperplastic changes of the NET cell may have the potential to evolve into neoplastic disease. This is particularly the case in the setting of MEN syndromes. Pseudohyperplasia of islet cells of the pancreatic head, however, is believed to be an age-dependent condition that differs from hyperplastic neoplastic NET disease [55]. The pancreas of patients with MEN1 (Fig. 2) typically contains multiple small NET-microadenomas [56]. It is supposed that the allelic loss of 11q13 in an islet cell sets the stage of NET development [57].

To our knowledge, no prospective study has so far specifically addressed the diagnostic performance with regard to the pancreas. The only information about the diagnostic performance of PET/CT has been derived from several retrospective studies using different sstr-avid tracers and protocols presented herein. Therefore, a standardised protocol is not yet available that especially addresses the knowledge about specificity of the technique. Are “FP findings” real non-tumour foci in PET/CT? What is the gold standard in this situation? Surgery? Follow-up? The uncertainty of this issue may also result in a patient tragedy [58].

Besides visual criteria to define abnormalities, the most attractive way to differentiate normal and abnormal pancreatic uptake might be based on quantitative parameters, in particular SUV_max_-values. However, the retrospective analyses show some discrepancies with regard to this parameter. For instance, we [22] found an SUV_max_ of 17.1 for ^68^Ga-DOTA-TOC that was best suited to discriminate between malignant and non-malignant lesions irrespective of the lesion diameter using ROC curves, while several other groups proposed somewhat different cut-off values. Extrapolating the data from the studies, the SUV_max_ cut-off for ^68^Ga-DOTA-TATE should be around 20 and for ^68^Ga-DOTA-NOC more than 20 [31–33]. While we initially could not demonstrate a relevant clinical value of SUV_max_ changes derived from ^68^Ga-DOTA-TOC studies in patients treated by PRRT [21], in a more recent publication Ambrosini et al. [59] recently demonstrated for ^68^Ga-DOTA-NOC that SUV_max_ is a relevant prognostic factor in G1- and G2-PNETs. In their retrospective analysis they showed for 43 patients that at the 24-month follow-up the SUV_max_ was significantly (*P* = 0.003) higher for patients with SD than for those with PD, reporting an SUV_max_ cut-off of 38.

However, some anecdotal examples show that even high SUV_max_ values can be found during repeated investigations without clinical evidence of tumour development, such as those shown here in Fig. 3. Therefore, cut-off values should be cautiously considered in the decision to perform surgery or not, and to specifically guide the patient management.

Summing up all available data, ^68^Ga-sstr-PET/CT imaging is feasible to rule out well-differentiated PNET in most cases with high reliability, which can be helpful in patients where the primary NET site is supposed in the pancreas. On the other hand, some papers report on the potential value of SUV_max_ calculation to assess NET tissue, however, this approach suffers from limitations mostly related to technical/methological shortcomings and sometimes the slow evolution of well-differentiated NETs. Therefore, prospective studies are required to standardise the procedure. Despite the frequent finding of enhanced uptake in the pancreatic head/uncinate process one should be cautious to assume this finding as normal to neglect the origin over time. In this situation at least another cross-sectional imaging modality, preferably DW-MRI, should be conducted to rule out any irregularity of the organ. Finally, it was already demonstrated that it is still possible that with a longer follow-up period some of the suggested foci that were “suspected tumours” and could not be verified by CT/MRI/EUS may be found to contain tumours in the future, such as recently demonstrated by Prasad et al. [28] or Etchebehere et al [40].

## Conclusion

On the basis of the present data, the diagnostic algorithm is to perform a ^68^Ga-sstr-PET/CT study on initial staging of the NET patient since it is superior to sstr-SPECT/CT as well as to DW-MRI. With regard to the pancreas, however, the definitive accuracy has yet to be defined. A huge scale of results can be observed to discriminate tumour from non-tumour findings by visual and in particular by quantitative scoring, from which it is not possible to make definite recommendations. The major limitation in most of these (single-centre) studies lies in the lack of histopathological confirmation of abnormal findings and the retrospective design. It must be mentioned that it is usually not feasible to obtain histopathological verification due to ethical and practical reasons, and therefore most of the published studies employed a combination of clinical and/or imaging follow-up.

## References

[1] Chua TC, Yang TX, Gill AJ, Samra JS (2016). Systematic review and meta-analysis of enucleation versus standardized resection for small pancreatic lesions. Ann Surg Oncol.

[2] Falconi M, Bartsch DK, Eriksson B (2012). ENETS Consensus guidelines for the management of patients with digestive neuroendocrine neoplasms of the digestive system: well-differentiated pancreatic non-functioning tumours. Neuroendocrinology.

[3] Hodul PJ, Strosberg JR, Kvols LK (2008). Aggressive surgical resection in the management of pancreatic neuroendocrine tumors: when is it indicated?. Cancer Control.

[4] Versari A, Camellini L, Carlinfante G (2010). Ga-68 DOTATOC-PET, endoscopic ultrasonography, and multidetector CT in the diagnosis of duodenopancreatic neuroendocrine tumors: a single-centre retrospective study. Clin Nucl Med.

[5] Bodei L, Sundin A, Kidd M, Prasad V, Modlin IM (2015). The status of neuroendocrine tumor imaging: from darkness to light?. Neuroendocrinology.

[6] Koopmans KP, Neels OC, Kema IP (2008). Improved staging of patients with carcinoid and islet cell tumors with ^18^F-dihydroxy-phenyl-alanine and ^11^C-5-hydroxy-tryptophan positron emission tomography. J Clin Oncol.

[7] Antwi K, Fani M, Nicolas G, Rottenburger C, Heye T, Reubi JC (2015). Localization of hidden insulinomas with ^68^Ga-DOTA-exendin-4 PET/CT: a pilot study. J Nucl Med.

[8] Reubi JC, Maurer R, von Werder K, Torhorst J, Klijn JG, Lamberts SW (1987). Somatostatin receptors in human endocrine tumors. Cancer Res.

[9] Virgolini I, Yang Q, Li S (1994). Cross-competition between vasoactive intestinal peptide and somatostatin for binding to tumor cell membrane receptors. Cancer Res.

[10] Malaisse-Lagae F, Stefan Y, Cox J, Perrelet A, Orci L (1979). Identification of a lobe in the adult human pancreas rich in pancreatic polypeptide. Diabetologia.

[11] Portela-Gomes GM, Stridsberg M, Grimelius L, Oberg K, Janson ET (2000). Expression of the five different somatostatin receptor subtypes in endocrine cells of the pancreas. Appl Immunohistochem Mol Morphol.

[12] Mandarino L, Stenner D, Blanchard W, Nissen S, Gerich J, Ling N (1981). Selective effects of somatostatin-14, -25 and -28 on in vitro insulin and glucagon secretion. Nature.

[13] Patel YC (1999). Somatostatin and its receptor family. Front Neuroendocrinol.

[14] Hofmann M, Maecke H, Börner R (2001). Biokinetics and imaging with the somatostatin receptor PET radioligand ^68^Ga-DOTATOC: preliminary data. Eur J Nucl Med.

[15] Reubi JC, Schär JC, Waser B (2000). Affinity profiles for human somatostatin receptor subtypes SST1-SST5 of somatostatin radiotracers selected for scintigraphic and radiotherapeutic use. Eur J Nucl Med.

[16] Antunes P, Ginj M, Zhang H (2007). Are radiogallium-labelled DOTA-conjugated somatostatin analogues superior to those labelled with other radiometals?. Eur J Nucl Med Mol Imaging.

[17] Wild D, Schmitt JS, Ginj M (2003). DOTA-NOC, a high-affinity ligand of somatostatin receptor subtypes 2, 3 and 5 for labelling with various radiometals. Eur J Nucl Med Mol Imaging.

[18] Kwekkeboom DJ, Kam BL, van Essen M (2010). Somatostatin-receptor-based imaging and therapy of gastroenteropancreatic neuroendocrine tumors. Endocr Relat Cancer.

[19] Virgolini I, Ambrosini V, Bomanji JB (2010). Procedure guidelines for PET/CT tumour imaging with ^68^Ga-DOTA-conjugated peptides: ^68^Ga-DOTA-TOC, ^68^Ga-DOTA-NOC, ^68^Ga-DOTA-TATE. Eur J Nucl Med Mol Imaging.

[20] Gabriel M, Decristoforo C, Kendler D (2007). ^68^Ga-DOTA-Tyr^3^-octreotide PET in neuroendocrine tumors: comparison with somatostatin receptor scintigraphy and CT. J Nucl Med.

[21] Gabriel M, Oberauer A, Dobrozemsky G (2009). ^68^Ga-DOTA-Tyr^3−^octreotide PET for assessing response to somatostatin-receptor-mediated radionuclide therapy. J Nucl Med.

[22] Kroiss A, Putzer D, Decristoforo C (2013). ^68^Ga-DOTA-TOC uptake in neuroendocrine tumour and healthy tissue: differentiation of physiological uptake and pathological processes in PET/CT. Eur J Nucl Med Mol Imaging.

[23] Al-Ibraheem A, Bundschuh RA, Notni J (2011). Focal uptake of ^68^Ga-DOTATOC in the pancreas: pathological or physiological correlate in patients with neuroendocrine tumours?. Eur J Nucl Med Mol Imaging.

[24] Kumar R, Sharma P, Garg P (2011). Role of ^68^Ga-DOTATOC PET-CT in the diagnosis and staging of pancreatic neuroendocrine tumours. Eur Radiol.

[25] Prasad V, Baum RP (2010). Biodistribution of the Ga-68 labeled somatostatin analogue DOTA-NOC in patients with neuroendocrine tumors: characterization of uptake in normal organs and tumor lesions. Q J Nucl Med Mol Imaging.

[26] Jacobsson H, Larsson P, Jonsson C, Jussing E, Grybäck P (2012). Normal uptake of ^68^Ga-DOTA-TOC by the pancreas uncinate process mimicking malignancy at somatostatin receptor PET. Clin Nucl Med.

[27] Castellucci P, Pou Ucha J, Fuccio C (2011). Incidence of increased ^68^Ga-DOTANOC uptake in the pancreatic head in a large series of extrapancreatic NET patients studied with sequential PET/CT. J Nucl Med.

[28] Prasad V, Ambrosini V, Hommann M, Hoersch D, Fanti S, Baum RP (2010). Detection of unknown primary neuroendocrine tumours (CUP-NET) using ^68^Ga-DOTA-NOC receptor PET/CT. Eur J Nucl Med Mol Imaging.

[29] Krausz Y, Rubinstein R, Appelbaum L (2012). Ga-68 DOTA-NOC uptake in the pancreas: pathological and physiological patterns. Clin Nucl Med.

[30] Sharma P, Arora S, Dhull VS (2015). Evaluation of ^68^Ga-DOTANOC PET/CT imaging in a large exclusive population of pancreatic neuroendocrine tumors. Abdom Imaging.

[31] Poeppel TD, Binse I, Petersenn S (2011). ^68^Ga-DOTATOC versus ^68^Ga-DOTATATE PET/CT in functional imaging of neuroendocrine tumors. J Nucl Med.

[32] Kabasakal L, Demirci E, Ocak M (2012). Comparison of ^68^Ga-DOTATATE and ^68^Ga-DOTANOC PET/CT imaging in the same patient group with neuroendocrine tumours. Eur J Nucl Med Mol Imaging.

[33] Wild D, Bomanji JB, Benkert P (2013). Comparison of ^68^Ga-DOTANOC and ^68^Ga-DOTATATE PET/CT within patients with gastroenteropancreatic neuroendocrine tumors. J Nucl Med.

[34] Skoura E, Michopoulou S, Mohmaduvesh M (2016). The impact of ^68^Ga-DOTATAE PET/CT imaging on management of patients with neuroendocrine tumours: experience from a national referral centre in the United Kingdom. J Nucl Med.

[35] Hofman MS, Kong G, Neels OC, Eu P, Hong E, Hicks RJ (2012). High management impact of Ga-68 DOTATATE (GaTate) PET/CT for imaging neuroendocrine and other somatostatin expressing tumours. J Med Imaging Radiat Oncol.

[36] Kunikowska J, Królicki L, Pawlak D, Zerizer I, Mikołajczak R (2012). Semiquantitative analysis and characterization of physiological biodistribution of ^68^Ga-DOTA-TATE PET/CT. Clin Nucl Med.

[37] Haug AR, Cindea-Drimus R, Auernhammer CJ (2012). The role of ^68^Ga-DOTATATE PET/CT in suspected neuroendocrine tumors. J Nucl Med.

[38] Ilhan H, Fendler WP, Cyran CC (2015). Impact of ^68^Ga-DOTATATE PET/CT on the surgical management of primary neuroendocrine tumors of the pancreas or ileum. Ann Surg Oncol.

[39] Schmid-Tannwald C, Schmid-Tannwald CM, Morelli JN (2013). Comparison of abdominal MRI with diffusion-weighted imaging to ^68^Ga-DOTATATE PET/CT in detection of neuroendocrine tumors of the pancreas. Eur J Nucl Med Mol Imaging.

[40] Etchebehere EC, de Oliveira SA, Gumz B (2014). ^68^Ga-DOTATATE PET/CT, ^99m^Tc-HYNIC-octreotide SPECT/CT, and whole-body MR imaging in detection of neuroendocrine tumors: a prospective trial. J Nucl Med.

[41] Putzer D, Kroiss A, Waitz D (2013). Somatostatin receptor PET in neuroendocrine tumours: ^68^Ga-DOTA^0^, Tyr^3^-octreotide versus ^68^Ga-DOTA^0^-lanreotide. Eur J Nucl Med Mol Imaging.

[42] Pfeifer A, Knigge U, Mortensen J (2012). Clinical PET of neuroendocrine tumors using ^64^Cu-DOTATATE: first-in-humans study. J Nucl Med.

[43] Krenning EP, Kwekkeboom DJ, Bakker WH (1993). Somatostatin receptor scintigraphy with [111In-DTPA-D-Phe1]- and [123I-Tyr3]-octreotide: the Rotterdam experience with more than 1000 patients. Eur J Nucl Med.

[44] Virgolini I, Innsbruck Team (2015). Peptide receptor radionuclide therapy (PRRT): clinical significance of re-treatment?. Eur J Nucl Med Mol Imaging.

[45] Wild D, Maecke HR, Waser B (2005). ^68^Ga-DOTANOC: a first compound for PET imaging with high affinity for somatostatin receptor subtypes 2 and 5. Eur J Nucl Med Mol Imaging.

[46] Kumar U, Sasi R, Suresh S (1999). Subtype-selective expression of the five somatostatin receptors (hSSTR1-5) in human pancreatic islet cells: a quantitative double-label immunohistochemical analysis. Diabetes.

[47] Wittingen J, Frey CF (1974). Islet concentration in the head, body, tail and uncinate process of the pancreas. Ann Surg.

[48] Miederer M, Seidl S, Buck A (2009). Correlation of immunohistopathological expression of somatostatin receptor 2 with standardised uptake values in ^68^Ga-DOTATOC PET/CT. Eur J Nucl Med Mol Imaging.

[49] Velikyan I, Sundin A, Sörensen J (2014). Quantitative and qualitative intrapatient comparison of ^68^Ga-DOTATOC and ^68^Ga-DOTATATE: net uptake rate for accurate quantification. J Nucl Med.

[50] Geworski L, Knoop BO, de Wit M, Ivancević V, Bares R, Munz DL (2002). Multicenter comparison of calibration and cross calibration of PET scanners. J Nucl Med.

[51] Scheuermann JS, Saffer JR, Karp JS, Levering AM, Siegel BA (2009). Qualification of PET scanners for use in multicenter cancer clinical trials: the American College of Radiology Imaging Network experience. J Nucl Med.

[52] Velikyan I, Sundin A, Eriksson B (2010). In vivo binding of [68Ga]-DOTATOC to somatostatin receptors in neuroendocrine tumours--impact of peptide mass. Nucl Med Biol.

[53] Winter A, Bundschuh RA, Al-Ibraheem A, Buck A, Schwaiger M, Scheidhauer K (2009). Ga-68-DOTATOC PET/CT - potential pitfalls in the volume of the upper abdomen. Eur J Nucl Med Mol Imaging.

[54] Klöppel G, Anlauf M, Perren A, Sipos B (2014). Hyperplasia to neoplasia sequence of duodenal and pancreatic neuroendocrine diseases and pseudohyperplasia of the PP-cells in the pancreas. Endocr Pathol.

[55] Anlauf M, Schlenger R, Perren A (2006). Microadenomatosis of the endocrine pancreas in patients with and without the multiple endocrine neoplasia type 1 syndrome. Am J Surg Pathol.

[56] Perren A, Anlauf M, Henopp T (2007). Multiple endocrine neoplasia type 1 (MEN1): loss of one MEN1 allele in tumors and monohormonal endocrine cell clusters but not in islet hyperplasia of the pancreas. J Clin Endocrinol Metab.

[57] Ambrosini V, Campana D, Polverari G (2015). Prognostic value of ^68^Ga-DOTANOC PET/CT SUVmax in patients with neuroendocrine tumors of the pancreas. J Nucl Med.

[58] Lois C, Jakoby BW, Long MJ (2010). An assessment of the impact of incorporating time-of-flight information into clinical PET/CT imaging. J Nucl Med.

[59] Lum S. Businessman looses lawsuit against cancer centre surgeon. The STRAIT TIMES. Febr 23, 2016, 5:00 AM, SGT. http://www.straitstimes.com/singapore/courts-crime/businessman-loses-lawsuit-against Accessed 23 Feb 2016.

